# The mitochondrial genome of a wild edible mushroom, *Russula rosea*

**DOI:** 10.1080/23802359.2022.2067502

**Published:** 2022-06-20

**Authors:** Fei Yu, Junfeng Liang

**Affiliations:** a College of Forestry, Shanxi Agricultural University, Taigu, China; b Key Laboratory of State Forestry Administration on Tropical Forestry Research, Research Institute of Tropical Forestry, Chinese Academy of Forestry, Guangzhou, China

**Keywords:** *Russula rosea*, mitogenomic, phylogenetic analysis

## Abstract

*Russula rosea* is a common wild edible ectomycorrhizal fungus, which is widely distributed all over the world. We assembled the complete mitochondrial genome of *R. rosea* with the total length was 54177 bp and the GC content of 22.34%. It contains a total of 57 genes, including 14 standard protein-coding genes, one conserved ribosomal protein S3 gene (*rps3*), two rRNA genes, 24 tRNA genes, 15 free-standing open reading frames (ORFs) and one DNA polymerase gene (*dpo*). Mitochondrial genome found a close evolutionary relationship between *Russula rosea* and *Russula lepida*, which was helpful to study the genetic evolutionary relationship of edible fungi.

*Russula rosea* (Pers. 1796) is one of the important wild edible ectomycorrhizal fungi in the world (Karliński et al. 2007, Kolmakov 2015), which also has antioxidant, anti-tumor and other medicinal effects (Kostić et al. 2020). It is a functional food with high protein, rough fiber and low fat with high nutritional and economic value (Karliński et al. 2007). Mitochondria, as an important organelle of eukaryotic cells, have their own genome and genetic mechanism, and play an important role in cell energy metabolism and biosynthesis, activation of drug resistance (Sandor et al. 2018). The mitochondrial genome has contributed to system evolution and population genetics (Li et al. 2018), but the mitochondrial genome of *R. rosea* not been described. In this study, we sequenced and annotated the mitochondrial genome of *R. rosea* to provide theoretical basis for phylogenetic relationships.

Fruiting bodies of *R. rosea* (strain F10) (Figure 1(A)) were collected from Huangshan, Anhui Province, China (118°18′ E, 30°9′ N), collected and identified by Fei Yu (email: yufei_1007@163.com). The materials were collected according to the guidelines of China, Anhui Province and the Research Institute of Tropical Forestry, Chinese Academy of Forestry, while the voucher specimen (RITF5754) was stored in the Research Institute of Tropical Forestry, Chinese Academy of Forestry, Guangzhou City, Guangdong Province, China (http://ritf.caf.ac.cn/, Junfeng Liang, jfliang2000@163.com). *R. rosea* fruiting bodies were divided into two fractions: one fraction was stored in a − 80 °C refrigerator, and the other fraction was 50 °C dried brought back to the laboratory. The specimen genomic DNA was extracted by Omega Fungal DNA Kit D3390-02, and species identification was performed, then genomic sequenced using Illumina NovaSeq 6000 at Novogene Bioinformatic Technology Co., Ltd, China.

**Figure 1.**
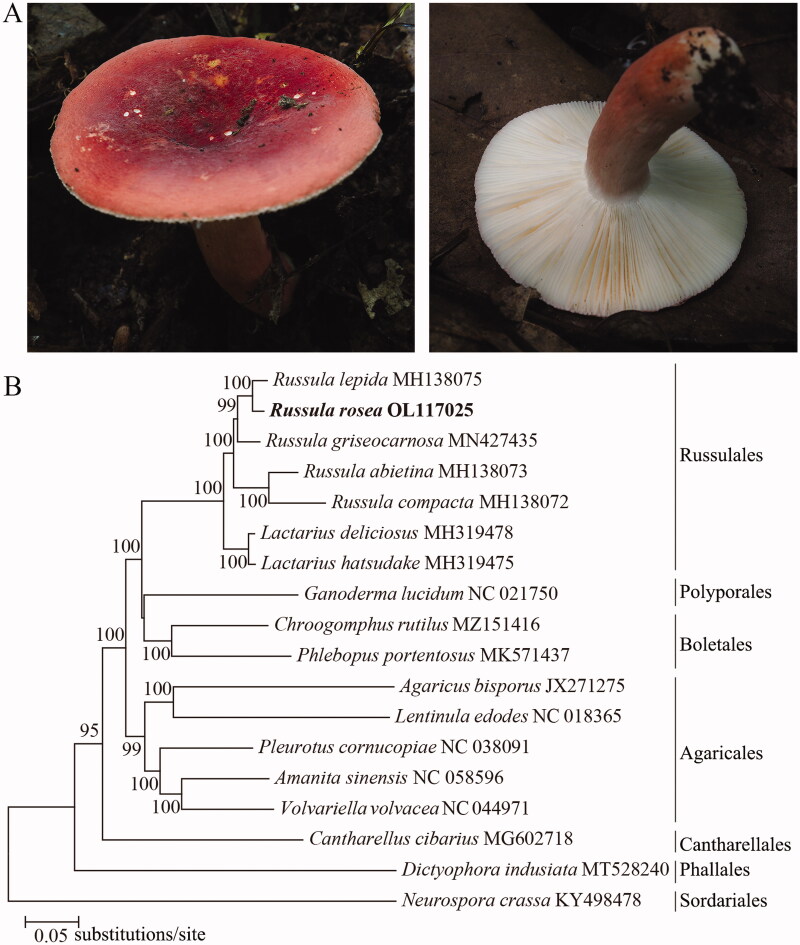
Phylogenetic tree (A) *Russula rosea*; (B) Phylogenetic tree of *R.rosea and* 18 fungi based on 14 standard protein coding genes.

The assembly of the mitogenomic of *R.rosea* were carried out using GetOrganelle v 1.7.5 (Jin et al. 2020), which based on bowtie2 v 2.3.5.1 (Langmead and Salzberg 2012) map reads to fungal mitogenomes (fungus_mt) database in NCBI and producted the de novo assembly using SPAdes v 3.13.0 (Bankevich et al. 2012), eventually generating the complete circular mitogenomes. The K-mer gradient was set to ‘-k 21, 45, 65, 85,105’ according to the sequenced read length of 150 bp. The mitogenomic was automatically annotated with online tool MFannot (Valach et al. 2014) based on mold mitochondrial genetic code (genetic code 4) (Li et al. 2018). Open Reading Frames (ORFs) were corrected using the online tool NCBI open reading frame finder, tRNA genes were predicted using online tRNAscan-SE 2.0 (Lowe and Chan 2016), and then perform a manual check to correct possible errors.

The mitogenomic of *R. rosea* was assembled as a 54177-bp circular molecule with a GC content of 22.34% (GenBank accession No. OL117025). It contains 57 genes, including 14 protein-coding genes (*atp6*, *atp8*, *atp9*, *cob*, *cox1*, *cox2*, *cox3*, *nad1*, *nad2*, *nad3*, *nad4*, *nad4L*, *nad5* and *nad6*), one conserved ribosomal protein S3 gene (*rps3*), two rRNA genes (*rns* and *rnl*), 24 tRNA genes, 15 free-standing open reading frames (ORFs) and one DNA polymerase gene (*dpo*). The initiation codon of 14 protein coding genes and conserved ribosomal protein S3 gene were ATG and the termination codon were TAA. The *R. rosea* mitogenome contains three tRNA genes with two different anticodons for arginine, leucine and serine, and two tRNA genes with the same anticodons for methionine. The mitogenome of *R. rosea* contains nine introns, which were located at *cob* (2 introns), *cox1* (4 introns), *cox2* (1 introns), *nad1* (1 intron) and *nad5* (1 intron). The overall nucleotide composition was A: 39.01%, T: 38.65%, C: 10.43%, and G: 11.91%.

To understand the evolution of *R. rosea*, we selected the mitochondrial genomes of 17 edible fungi published on NCBI website, took *Neurospora crassa* as outgroup, aligned the sequences of 14 protein-coding genes respectively, then concatenated alignments with SequenceMatrix v1.8 (Li et al. 2018), and finally constructed a neighbor-joining phylogenetic tree with Mega v7 by using 1000 bootstrap replicates (Figure 1(B)). Based on the neighbor-joining phylogenetic tree constructed by the nucleotide sequences of 14 protein coding genes, it was found that there was a close genetic relationship between *Russula rosea* and *Russula lepida*.

## Data Availability

The genome sequence data that support the findings of this study are openly available in GenBank of NCBI at https://www.ncbi.nlm.nih.gov under the accession no. OL117025. The associated BioProject, BioSample, and SRA numbers are PRJNA800019, SAMN25224323 and SRR17717762, respectively.
